# Dietary resveratrol improves antioxidant status of sows and piglets and regulates antioxidant gene expression in placenta by Keap1-Nrf2 pathway and Sirt1

**DOI:** 10.1186/s40104-018-0248-y

**Published:** 2018-04-20

**Authors:** Qingwei Meng, Tao Guo, Gaoqiang Li, Shishuai Sun, Shiqi He, Baojing Cheng, Baoming Shi, Anshan Shan

**Affiliations:** Institute of Animal Nutrition, Northeast Agricultural University, Harbin, Heilongjiang People’s Republic of China

**Keywords:** Nrf2, Oxidative stress, Piglet, Placenta, Resveratrol, Sirt1, Sow

## Abstract

**Background:**

Resveratrol, a plant phenol, affords protection against inflammation and oxidative stress. The objective of this study was to investigate the effects of dietary resveratrol supplementation during pregnancy and lactation on the antioxidant status of sows and piglets and on antioxidant gene expression and pathway in placenta.

**Methods:**

Forty sows were allotted to 2 dietary treatments 20 d after breeding. Sows were fed a control diet and a control diet with 300 mg/kg resveratrol. Oxidative stress biomarkers and antioxidant enzymes were measured in the placenta, milk, and plasma of sows and piglets. Antioxidant gene expression and protein expression of Kelch-like ECH-associated protein 1-Nuclear factor E2-related factor 2 (Keap1-Nrf2), nuclear factor kappa B-p65 (NFκB-p65) and sirtuin1 (Sirt1) were quantified in the placenta.

**Results:**

Dietary resveratrol increased the litter and piglets weaning weights. Antioxidant status in the milk, placenta and plasma of sows and piglets was partially improved by dietary resveratrol. In placenta, Nrf2 protein expression was increased and Keap1 protein expression was decreased by dietary resveratrol. The mRNA expression of antioxidant genes including catalase (*CAT*), glutathione peroxidase 1 (*GPX1*), *GPX4*, superoxide dismutase 1 (*SOD1*) and heme oxygenase 1 (*HO1*), and phase 2 detoxification genes, including glutamate-cysteine ligase modifier (*GCLM*), microsomal glutathione S-transferase 1(*MGST1*) and UDP glucuronosyltransferase family 1 member A1 (*UGT1A1*), was increased by dietary resveratrol. Dietary resveratrol also increased Sirt1 and phosphorylated NFκB-p65 protein expression in the placenta. We failed to observe any influences of dietary resveratrol on pro-inflammatory cytokine levels, including those of interleukin 1β (IL-1β), IL-6, IL-8 and tumor necrosis factor α (TNF-α). However, we observed that the mRNA expression of *IL-8* in placenta was reduced by maternal resveratrol. In addition, dietary resveratrol showed interactive effects with day of lactation on activities of SOD and CAT and levels of malonaldehyde (MDA) and hydrogen peroxide (H_2_O_2_) in milk.

**Conclusions:**

Dietary resveratrol supplementation during pregnancy and lactation improves the antioxidant status of sows and piglets, which is beneficial to the reproductive performance of sows. Dietary resveratrol regulates placental antioxidant gene expression by the Keap1-Nrf2 pathway and Sirt1 in placenta.

## Background

Oxidative stress, an imbalance between pro-oxidative and anti-oxidative forces in biological systems, may cause both lipid and protein oxidation and impair normal cell function. This imbalance is considered to be responsible for the initiation or development of pathological processes affecting female reproductive processes [1, 2]. Studies have reported that lipid peroxidation and oxidative stress are higher during normal pregnancy than during non-pregnancy in women [3]. During late gestation and lactation of sows, increasing rates of digestion, absorption, and tissue mobilization for fetal growth, mammary development and milk production favor overproduction of reactive oxygen species (ROS) and may cause oxidative stress in the maternal body. In addition, studies have confirmed that sows suffer elevated and systemic oxidative stress during late gestation and lactation [4, 5]. Berchieri-Ronchi et al. [6] observed elevated DNA damage throughout gestation and lactation compared with that observed during early gestation, and found that plasma α-tocopherol concentrations were lower at d 110 of gestation than during the lactation period in highly prolific sows. Tan et al. [5] reported that sows suffered increased oxidative stress during delivery, late gestation and early lactation, as indicated by their elevated ROS, 8-hydroxy-deoxyguanosine, and thiobarbituric acid reactive substance levels. In addition, oxidative stress has been suggested as a causative agent in human and animal pregnancy-related disorders, such as embryonic reabsorption, recurrent pregnancy loss, preeclampsia, intra-uterine growth restriction and fetal death, which are predictive of an elevated risk of the metabolic syndrome in postnatal life and may be a common pathway in developmental metabolic programming [7, 8].

Intake of dietary antioxidant nutrients can improve the endogenous antioxidant defense capacity, which has been considered a plausible way to prevent oxidative stress. In sows, accumulating studies have demonstrated that dietary antioxidant nutrients, such as vitamin E, vitamin C and selenium, improve antioxidant defense capacity and alleviate oxidative stress effectively, which are beneficial to litter size and piglet growth [9–11]. Polyphenols naturally occurring in plants, including flavonoids and phenolic acids have been demonstrated powerful antioxidant activities in vitro and in vivo [12]. Resveratrol is a polyphenol found in grapes, berries, peanuts and herbal medicines, and belongs to the stilbene family of phytoalexins, which are antibiotic compounds produced by plants in response to infection [13]. Resveratrol has generated intense scientific and public interest in recent years, mainly because of its widely reported ability to delay aging and prevent age-related diseases [14, 15]. Furthermore, resveratrol intake exerts significant beneficial effects in the treatment of cancer, type 2 diabetes, and cardiovascular and neurodegenerative diseases [16–19]. One of the biological activities that have been ascribed to resveratrol is its antioxidant potential. Resveratrol exerts a strong inhibitory effect on the production of ROS and free-radical-scavenging properties in many experimental systems [20–22]. Studies have shown that administration of resveratrol during pregnancy prevents oxidative stress and apoptosis in embryos of diabetic dams and relieves low-protein-diet-induced maternal, placental and offspring oxidative stress and metabolic dysfunction [23, 24]. Resveratrol has been used as a therapeutic agent for pregnancy complications in rodent models, such as preeclampsia [25], fetal growth restriction [26] and gestational diabetes [27]. To our knowledge, there are no available data assessing the effect of dietary resveratrol supplementation during gestation and lactation on the reproductive performance and antioxidant status of sows. Therefore, we tested the hypothesis that dietary resveratrol supplementation during gestation and lactation of sows improves the antioxidant status of sows and piglets, which may be beneficial to reproductive performance. We also hypothesized that the antioxidant gene and pathway may be regulated by dietary resveratrol.

## Methods

### Animals and management

Forty sows (Yorkshire, average parity 4.4) were bred with semen from a pool of Landrace boars. Forty sows were allotted to 2 dietary treatments 20 d after breeding. The treatment groups were as follows: 1) control sows fed a corn-soybean meal control diet (Con treatment, *n* = 20) and 2) treatment sows fed a control diet with 300 mg/kg resveratrol for 20 d after breeding through gestation and lactation (Res treatment, *n* = 20). The sows were kept in single crates (0.6 m × 2.0 m) from insemination to d 110 of gestation. On d 110 of gestation, sows were transported to the farrowing facility, where they were placed in individual farrowing crates (2.4 m × 2.4 m). Each crate had steel mesh floors with a heat lamp for newborn pigs. The crates were mounted over a solid concrete floor, and manure was removed manually each day. The farrowing room temperature was maintained at approximately 18 to 20 °C. Parturitions were watched but observers interfered as little as possible in the farrowing process. Within 24 h of birth, the piglets were cross-fostered within dietary treatment groups to standardize the litter size to range from 9 to12 piglets. At d 7, piglets received an iron injection, and males were castrated. The piglets were weaned at d 21 of lactation. The protocols used in this experiment were approved by the Northeast Agricultural University Institutional Animal Care and Use Committee. All animal experimental procedures were approved by the Ethical and Animal Welfare Committee of Heilongjiang Province, China.

### Experimental diets and feeding

Experimental diets (Table 1) were formulated to meet or exceed the recommended nutrient requirements of swine [28]. From d 20 of gestation until d 90 of gestation, all sows were fed 2.5 kg of the gestation diet daily. From d 91 of gestation, all sows were fed 3.5 kg of the gestation diet daily. The amounts of parturition feed provided to each sow at d 112, 113 and 114 of gestation were 2.0, 1.5 and 1.0 kg, respectively. On the day of farrowing, sows were offered 2.0 kg of the lactation diet. Thereafter, this amount was increased by 1.0 kg daily until ad libitum feeding.

**Table 1:** Composition and nutrient levels of diets (as-fed basis)

Item	Gestation	Lactation
Ingredient, %		
Corn	66.0	62.8
Soybean meal	15.0	24.0
Wheat bran	16.0	4.0
Fish meal	–	3.0
Soybean oil	–	3.0
Dicalcium phosphate	1.0	1.3
Limestone	1.1	0.8
*L*-Lysine HCl (78%)	–	0.2
Salt	0.4	0.4
Premix^1^	0.5	0.5
Nutritional composition^2^, %		
Net energy, MJ/kg	9.46	10.38
Crude protein (CP)	14.03	18.28
Calcium	0.71	0.82
Total phosphorus	0.60	0.68
Available phosphorus	0.31	0.43
SID Lysine	0.54	0.99

^1^The premix provides following for per kg diet: vitamin A, 8,000 U; vitamin D_3_, 2,000 U; vitamin E, 50 U; vitamin K_3_, 1.5 mg; vitamin B_1_, 1.6 mg; vitamin B_6_, 1.5 mg; vitamin B_12_,15 μg; niacin, 20 mg; *D*-pantothenic acid,15 mg; Zn (ZnO), 100 mg; Fe (FeSO_4_·7H_2_O), 80 mg; Cu (CuSO_4_·5H_2_O), 20 mg; Mn (MnSO_4_·H_2_O), 25 mg; I (KI), 0.3 mg; Se (NaSeO_3_·5H_2_O), 0.2 mg

^2^Nutrient levels were calculated values

### Sample and data collection

Blood was collected from the ear vein of a random subset of sows (*n* = 8 per treatment) at d 110 of gestation and at d 14 and 21 of lactation. The same subset of sows was bled at each time interval. In addition, blood samples were collected from new born pigs (*n* = 8 per treatment, 1 piglet per litter) and weaning piglets (*n* = 8 per treatment, 1 piglet per litter) by vena puncture. The new born piglets selected for blood collecting were removed from their mothers immediately after birth without sucking colostrum. The blood was centrifuged at 3,000×*g* for 15 min to obtain the plasma, and stored at − 20 °C until analysis. Eight sows per treatment were randomly selected and marked for milk sample collection during lactation. Colostrum was collected within 6 h of farrowing (d 0 of lactation), and mature milk samples were collected at d 7, 14 and 21 of lactation. Approximately 30 to 50 mL of milk was collected from all functional mammary glands using a mechanical milk pump after injection of 1 mL oxytocin. The samples were immediately stored at − 20 °C until analysis. Placenta allantochorion tissue samples were collected immediately during parturition to preserve RNA stability for mRNA analysis. A section of samples was stored at − 20 °C, and another section was snap-frozen in liquid nitrogen for further analysis.

Sow back-fat thickness was measured at d 20 and 110 of gestation, within 24 h of farrowing (d 0) and d 21 of lactation. Back-fat thickness was measured at the P2 position (left side of the 10^th^ rib and 6 cm lateral to the spine) by digital B-ultrasonography (Kaixin, Xuzhou, China). At farrowing, the number of piglets born, litter birth weight and individual birth weights were recorded. At weaning, the number of pigs weaned, litter weight and individual pig weight were measured.

### Evaluation of antioxidant enzyme activity

Superoxide dismutase (SOD), glutathione peroxidase (GSH-Px) and catalase (CAT) enzyme activities in plasma, placenta and milk were determined using commercially available kits (Nanjing Jiancheng Bioengineering Institute, Nanjing, China), in accord with our previous study [29]. The results of the measurements were expressed as U/mL in plasma and milk and as U/mg protein in placenta.

### Assessment of lipid peroxidation

Lipid peroxidation in plasma, placenta and milk was determined by measuring the amounts of malondialdehyde (MDA) through the thiobarbituric acid method using commercially available kits (Nanjing Jiancheng Bioengineering Institute, Nanjing, China). The results of the measurements were expressed as nmol/mL in plasma and milk and as nmol/mg protein in placenta.

### Assessment of hydrogen peroxide (H_2_O_2_)

Hydrogen peroxide (H_2_O_2_) was determined by a commercially available kit (Nanjing Jiancheng Bioengineering Institute, Nanjing, China). H_2_O_2_ bound with molybdenic acid to form a complex, which was measured at 405 nm. The results of the measurements were expressed as mmol/L in plasma and milk and as mmol/g protein in placenta.

### Assessment of pro-inflammatory cytokine

Placenta tissue homogenates (10%) were analyzed for pro-inflammatory cytokine content. Commercial ELISA kits were used to detect the interleukin 1β (IL-1β), IL-6, IL-8, and tumor necrosis factor α (TNF-α) levels in placenta (Cusabio, Wuhan, China). The results were expressed as pg/mg protein.

### Real-time PCR (RT-PCR)

Total RNA was extracted from approximately 100 mg of frozen placenta tissues using the reagent box of Total RNA Kit according to the manufacturer’s instructions. The concentration of RNA was measured using a spectrophotometer, and the purity was ascertained by the A260/A280 ratio. The quality of RNA was evaluated by agarose gel electrophoresis. Total RNA (2 μg) from each sample was converted to cDNA for RT-PCR using the Prime Script RT reagent Kit (TaKaRa Bio Catalog). RT-PCR was performed using the SYBR Green I Kit (TaKaRa Bio Catalog). For analyses on an ABI PRISM 7500 SDS thermal cycler, PCR reactions were performed with 2.0 μL of first-strand cDNA and 0.4 μL of sense and anti-sense primers in a final volume of 20 μL. Samples were centrifuged briefly and run on the PCR machine using the default fast program (1 cycle at 95 °C for 30 s, 40 cycles of 95 °C for 5 s and 60 °C for 34 s). All PCR reactions were performed in triplicate. Pig-specific primers were designed from published GenBank sequences and were synthesized by Sangon Biotech (Table 2). The specificity of primers was examined by Primer-BLAST tool (https://www.ncbi.nlm.nih.gov/tools/primer-blast) and confirmed by single peaks in the dissociation curves. *GAPDH* was used as an internal control gene, as it did not respond to dietary treatments. Data were obtained as Ct values (cycle number at the threshold). The relative gene expression levels were calculated using the 2^-ΔΔCt^ method, normalizing to *GAPDH* expression [30].

**Table 2:** Primers used for Real-time PCR

Genes	Sequence (5’to3’)	Product size, bp	GenBank No.
*GPX4*	F: CACCCTCTGTGGAAGTGGAT	112	NM_214407.1
	R: TCACCACACAGCCGTTCTTA		
*GPX1*	F: AAATGCTCACCCGCTCTTC	118	NM_214201.1
	R: GTCATTGCGACACACTGGAG		
*CAT*	F: ACGCCTGTGTGAGAACATTG	124	NM_214301.2
	R: GTCCAGAAGAGCCTGAATGC		
*SOD3*	F: ACGCTGCTCTGTGCTTACCT	135	NM_001078688.1
	R: CTGCCAGATCTCCGTCACTT		
*SOD2*	F: TGGAGGCCACATCAATCATA	113	NM_214127.2
	R: TTTCGAAGGAACCAAAGTCG		
*SOD1*	F: TCCATGTCCATCAGTTTGGA	131	NM_001190422.1
	R: AGTCACATTGCCCAGGTCTC		
*GAPDH*	F: ATGGTGAAGGTCGGAGTGAA	155	NM_001206359.1
	R: CCGTGGGTGGAATCATACTG		
*NQO1*	F: CCAGCAGCCCGGCCAATCTG	160	NM_001159613.1
	R: AGGTCCGACACGGCGACCTC		
*HO1*	F: AGGCTGAGAATGCCGAGTTC	90	NM_001004027.1
	R: TGTGGTACAAGGACGCCATC		
*CYP1A1*	F: CTGCCATCTTCTGCCTTGTA	314	NM_214412.1
	R: GCTCTGGCCATTAGAGATCA		
*TXNRD1*	F: CTTTACCTTATTGCCCGGGT	162	NM_214154.2
	R: GTTCACCGATTTTGTTGGCC		
*GCLM*	F: CTTGCCTCTTGCTGTGTGAT	159	XM_001926378.4
	R: CCACTCATGTGCCTCGATGT		
*MGST1*	F: TTGGCGCGCGAATCTACCACA	239	NM_214300.1
	R: TCCTCGGCTCCCTTCCCACTTA		
*UGT1A1*	F: GATCCTTTCCTGCAACGCAT	313	XM_001927673
	R: GGAAGGTCATGTGATCTGAG		
*IL-6*	F: AGCAAGGAGGTACTGGCAGA	257	NM_001252429.1
	R: GTGGTGGCTTTGTCTGGATT		
*IL-8*	F: ACTTCCAAACTGGCTGTTGC	120	NM_213867.1
	R: GGAATGCGTATTTATGCACTGG		
*IL-1β*	F: GTTCTCTGAGAAATGGGAGC	143	NM_214055.1
	R: CTGGTCATCATCACAGAAGG		
*TNF-α*	CATGAGCACTGAGAGCATGA	170	NM_214022.1
	CGATAACTTCGAAGTGCAGT		

*GPx4*, glutathione peroxidase 4; *GPx1*, glutathione peroxidase 1; *CAT*, catalase; *SOD3*, superoxide dismutase 3; *SOD2*, superoxide dismutase 2; *SOD1*, superoxide dismutase 1; *NQO1*, NAD(P)H quinone dehydrogenase 1; *HO1*, heme oxygenase 1; *CYP1A1*, cytochrome P450 family 1 subfamily A member 1; *TXNRD1*, thioredoxin reductase 1; *GCLM*, glutamate-cysteine ligase modifier; *MGST1*, microsomal glutathione S-transferase 1; *UGT1A1*, UDP glucuronosyl- transferase family 1 member A1; *IL-1*, interleukin 1; *IL-6*, interleukin 6; *IL-8*, interleukin 8; *TNF-α*, tumor necrosis factor α

### Western blotting

Placental tissue was pulverized in liquid nitrogen and lysed in RIPA buffer (Beyotime Biotechnology, China) containing 1 mmol/L PMSF and 1% phosphatase inhibitor. After schizolysis for 1 h on ice, the extracts were centrifuged in a speed 12,000 r/min for 20 min at 4 °C. Then, the supernatant was obtained, and bicinchoninic acid assay was used to detect the protein concentration. Proteins (50 mg per lane) were transblotted to polyvinylidene difluoride membranes in standard tris-glycine transfer buffer, pH 8.3, containing 0.5% SDS. After transfer, membranes were blocked for 1 h at room temperature in TBST (10 mmol/L Tris-HCl, pH 8.0, 150 mmol/L NaCl, 0.2% Tween-20) containing 5% non-fat milk powder and incubated overnight at 4 °C with 1:500 diluted primary antibodies, including GAPDH (Wanlei Biotechnology), Sirt1 (Wanlei Biotechnology), NFκB-p65 (Santa Cruz Biotechnology), phosphorylated NFκB-p65 (Ser536, Santa Cruz Biotechnology), Nrf2 (Abacm) and Keap1(Bioss) antibodies. Membranes were then washed in TBST three times. The 1:5,000 diluted secondary antibodies (Zhongshan Golden Bridge) were added, with incubation for 2 h at 37 °C, and the membrane was washed three times. Western blot images were quantified by measuring the intensity of correctly sized bands using Alpha Imager 2200 (Alpha Innotech Corporation, CA, USA), and all protein measurements were normalized to GAPDH.

### Statistical analysis

The data were analyzed by ANOVA and multiple comparisons with Tukey’s method using SPSS 18.0 (IBM-SPSS Inc., Chicago, Illinois, USA). Milk antioxidant status data were analyzed using 2-factor repeated-measures ANOVA, with the factors in the model consisting of dietary resveratrol supplementation, day of lactation and their interactions. The individual sow and her litter were used as the experimental unit. The results were presented as mean values and the standard error of the mean (SEM). Differences were considered significant if *P* ≤ 0.05 and a trend at *P* values between 0.05 and 0.10.

## Results

### Reproductive performance

The results pertaining to the reproductive performance of sows are shown in Table 3. The back-fat thickness of sows at d 20 and 110 of gestation and at weaning did not differ between the two dietary treatments (*P* > 0.05). Dietary resveratrol supplementation in gestation and lactation diets had no effects on back-fat thickness gain during gestation or loss during lactation (*P* > 0.05). The numbers of piglets born, piglets born alive and piglets weaned were not affected by dietary resveratrol (*P* > 0.05). Pre-weaning survival rate was not affected by dietary resveratrol supplementation during gestation or lactation (*P* > 0.05). The litter birth weight, piglet birth weight, born alive litter weight and born alive piglet weight were not influenced (*P* > 0.05) by dietary resveratrol. However, the litter weaning weight and piglet weaning weight were increased (*P* < 0.05) by dietary resveratrol during gestation and lactation.

**Table 3:** Effects of dietary resveratrol supplementation during gestation and lactation on reproductive performance of sows

Item	Con		Res		*P*-value
	Mean	SEM	Mean	SEM	
Sow back-fat thickness, mm					
Gestation (d 20)	14.44	0.87	14.22	0.99	0.867
Gestation (d 110)	17.35	1.26	17.43	1.44	0.968
Gain	2.91	0.39	3.21	0.47	0.623
Parturition	17.47	1.29	17.58	0.35	0.956
Weaning	15.80	1.09	15.84	0.95	0.976
Loss	1.68	0.29	1.74	0.49	0.915
Reproductive performance					
Number of piglets born	12.05	0.62	13.00	0.69	0.311
Litter birth weight, kg	17.59	0.82	18.52	0.67	0.388
Piglet birth weight, kg	1.48	0.06	1.47	0.07	0.898
Number of piglets born alive	11.32	0.60	11.94	0.54	0.444
Born alive litter weight, kg	16.71	0.80	17.52	0.54	0.410
Born alive piglet weight, kg	1.51	0.06	1.51	0.07	0.979
Number of piglets weaned	9.42	0.26	9.61	0.32	0.647
Litter weaning weight, kg	48.98	1.46	57.26	2.44	0.006
Piglet weaning weight, kg	5.24	0.12	5.84	0.20	0.015
Pre-weaning survival rate, %	84.79	0.03	83.00	0.03	0.626

Con Control treatment; Res Resveratrol treatment. All values are expressed as means and SEM (*n* = 20)

### Antioxidant status in plasma of sows and piglets

**Fig. 1:**
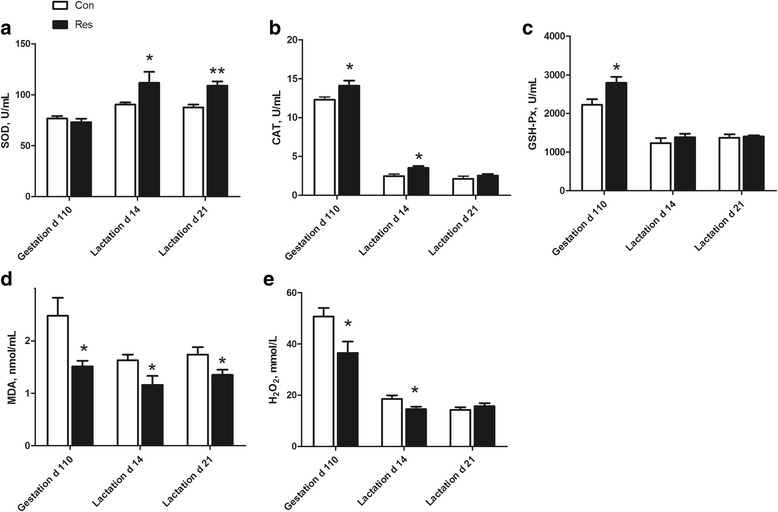
Effects of dietary resveratrol supplementation during gestation and lactation on antioxidant status in plasma of sows. Con, control treatment; Res, resveratrol treatment; **a** SOD, superoxide dismutase; **b** CAT, catalase; **c** GSH-Px, glutathione peroxidase; **d** MDA, malondialdehyde; **e** H_2_O_2_, hydrogen peroxide. All values are expressed as means ± SEM (*n* = 8). *P < 0.05, **P < 0.01

The results of antioxidant status in plasma of sows are presented in Fig. 1a-e. On d 14 (*P* = 0.042) and 21 (*P* = 0.001) of lactation, the SOD activity in the plasma of sows was increased by resveratrol supplementation. On d 110 of gestation, dietary supplementation of resveratrol increased (*P* < 0.05) the GSH-Px activity in sow plasma. The CAT activity in plasma of sows on d 110 of gestation and on d 14 of lactation was increased (*P* < 0.05) by resveratrol supplementation. The MDA level in the plasma of sows from the Res treatment was lower than that in sows from the Con treatment on d 110 of gestation and d 14 and 21 of lactation (*P* < 0.05). On d 110 of gestation and d 14 of lactation, the H_2_O_2_ level in the plasma of sows was decreased by dietary resveratrol supplementation (*P* < 0.05).

**Fig. 2:**
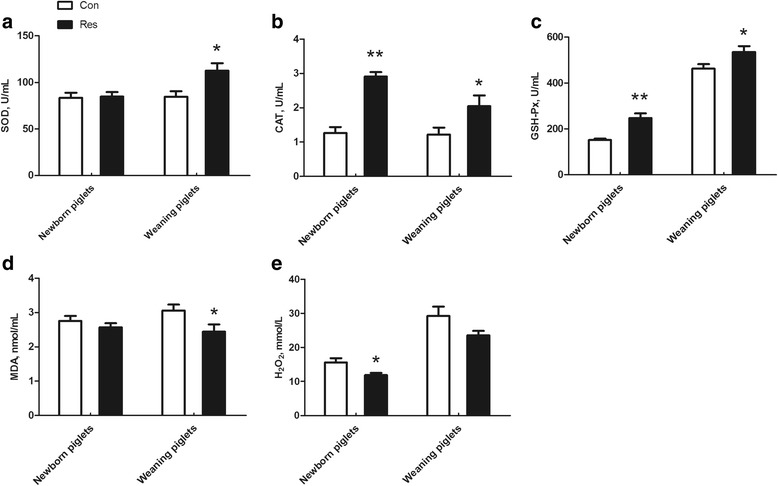
Effects of dietary resveratrol supplementation during gestation and lactation of sows on antioxidant status in plasma of piglets. Con, control treatment; Res, resveratrol treatment; **a** SOD, superoxide dismutase; **b** CAT, catalase; **c** GSH-Px, glutathione peroxidase; **d** MDA, malondialdehyde; **e** H_2_O_2_, hydrogen peroxide. All values are expressed as means ± SEM (*n* = 8). *P < 0.05, **P < 0.01

The GSH-Px and CAT activities in the plasma of new born piglets were increased (*P* < 0.01) by maternal dietary resveratrol supplementation (Fig. 2a-e). The H_2_O_2_ level in the plasma of newborn piglets was reduced by maternal dietary resveratrol supplementation (*P* < 0.05). The SOD activity and MDA level in the plasma of new piglets did not differ between the Con and Res treatments (*P* > 0.05). Dietary resveratrol supplementation during gestation and lactation increased (*P* < 0.05) the GSH-Px, SOD and CAT activities (*P* < 0.05), and reduced (*P* = 0.042) the MDA level in the plasma of weaning piglets. The H_2_O_2_ level in the plasma of weaning piglets tended to be reduced by maternal resveratrol supplementation (*P* = 0.09).

### Antioxidant status and pro-inflammatory cytokines in placenta

**Fig. 3:**
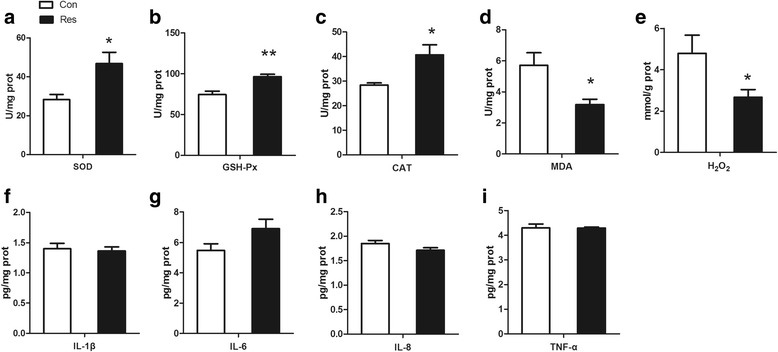
Effects of dietary resveratrol supplementation during gestation and lactation of sows on antioxidant status and pro-inflammatory cytokine in placenta. Con, control treatment; Res, resveratrol treatment; **a** SOD, superoxide dismutase; **b** GSH-Px, glutathione peroxidase; **c** CAT, catalase; **d** MDA, malondialdehyde; **e** H_2_O_2_, hydrogen peroxide; **f** IL-1β, interleukin 1β; **g** IL-6, interleukin 6; **h** IL-8, interleukin 8; **i** TNF-α, tumor necrosis factor α. All values are expressed as means ± SEM (*n* = 6). *P < 0.05, **P < 0.01

As shown in Fig. 3a-e, the SOD, GSH-Px and CAT activities in the placenta were increased (*P* < 0.05) by dietary resveratrol supplementation. The MDA level in the placenta was reduced (*P* = 0.022) by dietary resveratrol supplementation. In addition, dietary resveratrol supplementation decreased the H_2_O_2_ level in the placenta (*P* < 0.05). The levels of pro-inflammatory cytokines (Fig. 3f-i), including IL-1β, IL-8 and TNF-α, did not differ between the Con and Res treatments (*P* > 0.05). The content of IL-6 in the Res treatment tended (*P* = 0.091) to be higher than that in the Con treatment.

### Placental Sirt1, Keap1-Nrf2 and NFκB-p65 protein expression

**Fig. 4:**
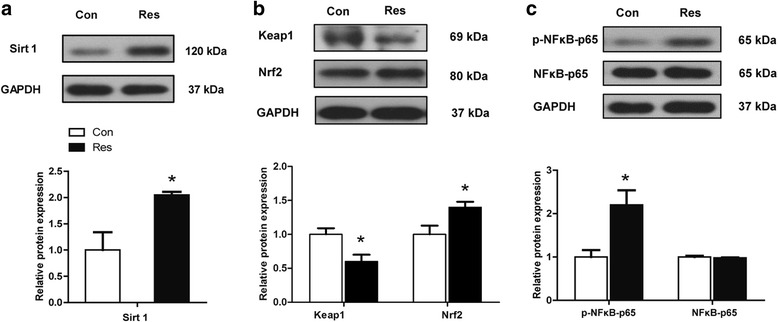
Effects of dietary resveratrol supplementation during gestation and lactation of sows on Sirt1, Nrf2-Keap1, NFκB-p65 and p-NFκB-p65 protein expression in placenta. Con, control treatment; Res, resveratrol treatment; **a** Sirt1 protein expression; **b** Keap1 and Nrf2 protein expression; **c** NFκB-p65 and p-NFκB-p65 (Ser536) protein expression; Con, control treatment; Res, resveratrol treatment; Nrf2, Nuclear factor E2-related factor 2; Keap1, Kelch-like ECH associated protein 1; NFκB, nuclear factor kappa B; p-NFκB, phosphorylated NFκB. All values are expressed as means ± SEM (*n* = 6). GAPDH was used as the internal control. *P < 0.05, **P < 0.01

As shown in Fig. 4, the protein expression of Sirt1 was increased (*P* < 0.05) by dietary resveratrol supplementation. Keap1 protein expression was decreased and Nrf2 protein expression was increased by dietary resveratrol supplementation (*P* < 0.05). The NFκB-p65 protein expression level did not differ (*P* > 0.05) between the Con and Res treatments but phosphorylated NFκB-p65 was increased (*P* = 0.037) by dietary resveratrol.

### Placental Nrf2- regulated gene expression

**Fig. 5:**
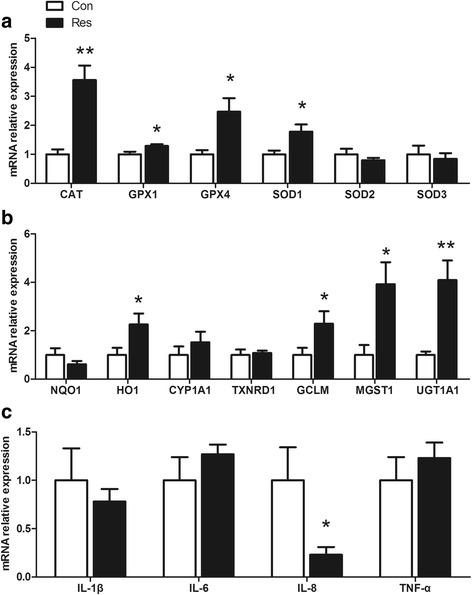
Effects of dietary resveratrol supplementation during gestation and lactation of sows on mRNA relative expression of Nrf2-regulated genes and pro-inflammatory cytokines in placenta. **a**, **b** mRNA relative expression of Nrf2-regulated genes; **c** mRNA relative expression of pro-inflammatory cytokines. Con, control treatment; Res, resveratrol treatment; *SOD*, superoxide dismutase; *Gpx*, glutathione peroxidase; *CAT*, catalase; *NQO1*, NAD(P)H quinone dehydrogenase 1; *HO1*, heme oxygenase 1; *CYP1A1*, cytochrome P450 family 1 subfamily A member 1; *TXNRD1*, thioredoxin reductase 1; *GCLM*, glutamate-cysteine ligase modifier; *MGST1*, microsomal glutathione S-transferase 1; *UGT1A1*, UDP glucuronosyl-transferase family 1 member A1; *IL-1β*, interleukin 1β; *IL-6*, interleukin 6; *IL-8*, interleukin 8; *TNF-α*, tumor necrosis factor α. All values are expressed as means ± SEM (n = 6). *GAPDH* was used as the internal control. *P < 0.05, **P < 0.01

The mRNA expression levels of Nrf2-regulated genes are presented in Fig. 5a-b. The mRNA expression levels of *CAT*, *GPX1*, *GPX4* and *SOD1* in placenta were increased (*P* < 0.05) by dietary resveratrol supplementation. Resveratrol supplementation failed (*P* > 0.05) to affect the mRNA expression levels of *SOD2*, *SOD3*, *NQO1*, *CYP1A1* and *TXNRD1* in placenta. In addition, the mRNA expression of *HO1*, *GCLM*, *MGST1* and *UGT1A1* was increased (*P* < 0.05) by dietary resveratrol.

### Placental pro-inflammatory cytokines gene expression

The mRNA expression of pro-inflammatory cytokines, including *IL-6*, *TNF-α* and *IL-1β*, was not affected (*P* > 0.05) by dietary resveratrol (Fig. 5c). The mRNA expression of *IL-8* was reduced by dietary resveratrol (*P* = 0.044).

### Antioxidant status in milk

**Fig. 6:**
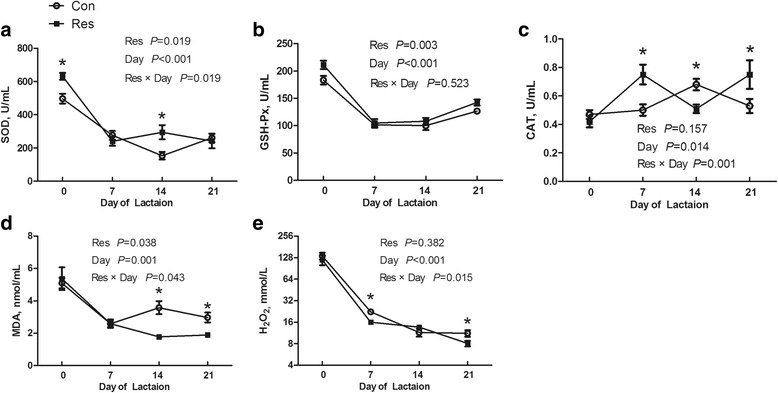
Effects of dietary resveratrol supplementation during gestation and lactation of sows on antioxidant status in milk. **a** SOD, superoxide dismutase; **b** GSH-Px, glutathione peroxidase; **c** CAT, catalase; **d** MDA, malondialdehyde; **e** H_2_O_2_, hydrogen peroxide. Con, control treatment; Res, resveratrol treatment; Day, day of lactation; Res × Day, interaction between Resveratrol and Day. All values are expressed as means ± SEM (*n* = 8). *P < 0.05, **P < 0.01

Interactive effects of dietary resveratrol and day of lactation were observed on activities of SOD (*P* = 0.019) and CAT (*P* = 0.001) and levels of MDA (*P* = 0.043) and H_2_O_2_ (*P* = 0.015) in milk (Fig. 6). SOD activity in milk from the Res sows was higher (*P* < 0.05) than that in milk from the Con sows at d 0 and 14 of lactation but did not (*P* > 0.05) differ between the Con and Res treatments at d 7 and 21 of lactation. Similarly, MDA level in milk did not (*P* > 0.05) differ between the Con and Res treatments at d 0 and 7 of lactation but was higher (*P* < 0.05) in milk from the Res sows at d 14 and 21 of lactation. The H_2_O_2_ level was reduced (*P* < 0.05) by dietary resveratrol at d 7 and 21 of lactation but not changed (*P* > 0.05) at d 0 and 14 of lactation. In addition, dietary resveratrol increased the CAT activity in milk at d 7 and 21 of lactation but decreased it at d 14 of lactation (*P* < 0.05). The GSH-Px activity in milk was increased by dietary resveratrol (*P* = 0.003). Overall, the activities of SOD (*P* < 0.001), CAT (*P* = 0.014) and GSH-Px (*P* < 0.001) and levels of MDA (*P* = 0.001) and H_2_O_2_ (*P* < 0.001) in milk were affected by day of lactation.

## Discussion

The major objective of the present study was to investigate the influence of dietary resveratrol supplementation during gestation and lactation on antioxidant status in sows and piglets and on antioxidant gene expression and pathway in placenta. To our knowledge, this is the first study to examine the effects of dietary resveratrol supplementation during gestation and lactation in sows. During pregnancy and lactation of sows, high energy and oxygen levels are required to satisfy increasing metabolic burdens for fetal growth, placenta development and milk production, which could cause elevated ROS production [4, 9]. In addition, studies have confirmed that sows suffer elevated systemic oxidative stress and DNA damage during late gestation and lactation [4, 5]. In normal human pregnancy, moderate oxidative stress levels have also been found, characterized by an increase in ROS biomarkers, which is balanced by an increase in biomarkers of the antioxidant system [3]. In our study, we measured antioxidant enzyme activities and oxidative stress markers (MDA and H_2_O_2_) in the plasma (sows and offspring), placenta and milk. We found that the antioxidant status of sows, including that in plasma, placenta and milk, was partially improved and oxidative stress markers were partially reduced by dietary resveratrol supplementation. In addition, the antioxidant status of piglets was partially increased by maternal resveratrol supplementation. Resveratrol has been shown to exhibit antioxidant, anti-inflammatory and antibacterial characteristics in human, rat, chicken and pig [13] and modulate multiple signaling pathways [17]. H_2_O_2_, a major type of ROS, is synthesized via a dismutation reaction catalyzed by SOD and reduced to water by GSH-Px or CAT [31]. H_2_O_2_ is also a molecular culprit to induced lipoperoxidation. MDA is the end product of lipoperoxidation, and serves as an excellent oxidative stress marker [23, 29]. In our study, dietary resveratrol supplementation increased antioxidant activity to a certain degree, and reduced the H_2_O_2_ and MDA levels, indicating that the oxidative stress in sows was reduced by dietary resveratrol supplementation. Our study is consistent with that of Vega et al. [23], who reported that dietary resveratrol reduced maternal oxidative stress biomarkers, including serum MDA and liver ROS, which were induced by low-protein diet.

Placenta, as the main interface between mother and fetus, is essential for ensuring optimal pregnancy and fetal outcomes, including appropriate organ development and fetal growth. Placental changes in oxygen tension help to sustain the increased metabolic rate that occurs during the rapid fetal growth phase and are associated with both increased circulating ROS and increased activity of enzymes; these responses help to protect against increased oxidative stress [32]. However, placenta is highly sensitive to oxidative stress [33]. In the present study, the antioxidant status of the placenta was improved, and the H_2_O_2_ and MDA levels in the placenta were decreased due to dietary resveratrol supplementation. To further explore the molecular mechanism by which dietary resveratrol affects oxidative stress status in placenta, the Keap1-Nrf2 signal pathway was analyzed.

Nrf2, a redox sensitive transcription factor, regulates intracellular antioxidants and phase II detoxification enzymes by the transcriptional activation of many genes containing antioxidant response elements (ARE) [34]. Keap1 has been identified as a cytosolic binding protein for Nrf2, which is associated with the Kelch domain of Keap1. Keap1 is sequestered in association with the actin cytoskeleton under normal physiological conditions, which in turn allows for proteasome degradation of Nrf2 [35]. In the present study, the Nrf2 protein expression level was increased and the Keap1 protein expression level was decreased, which indicated that the Nrf2 pathway was activated by dietary resveratrol supplementation. In addition, dietary resveratrol up-regulated the Nrf2-regulated genes involved in the antioxidant defense system, including *CAT*, *GPX1*, *GPX4*, *SOD1* and *HO1*. Dietary resveratrol also up-regulated genes related to phase II metabolism, such as *GCLM*, *MGST1* and *UGT1A1*. *HO1* contributes to the catalysis of heme to carbon monoxide, free ferrous iron and biliverdin [36]. *GCLM* is the modifier subunit of glutamate cysteine ligase, which catalyzes the rate-limiting reaction in the de novo synthesis of glutathione, an important antioxidant that contributes to the maintenance of cellular redox status [37]. Furthermore, the increased mRNA expression of the family of glutathione peroxidases (*GPX*), including *GPX1* and *GPX4*, is in accord with enzyme activity in placenta. Organisms with exogenous toxins have a certain ability to detoxify. The glutathione S-transferase (*GST*) and UDP-glucuronosyltransferase (*UGT*) families play an extremely important role in biological detoxification systems, which are modulated by Nrf2 and ARE [38]. *MGST1* and *UGT1A1*, phase II detoxification enzymes, were up-regulated, indicating that phase II detoxification was improved by dietary resveratrol supplementation. Resveratrol, initially characterized as a phytoalexin, can prevent or slow the progression of a wide variety of illnesses, including cancer, cardiovascular disease and ischemic injuries, as well as extend the lifespans of various organisms [17]. In our study, we demonstrated that dietary resveratrol during gestation can alleviate oxidative stress, and improve antioxidant capacity in placenta via the Nrf2 pathway. Moreover, the antioxidant gene expression regulated by Nrf2 was increased by dietary resveratrol.

Sirt1 is a nicotinamide adenine dinucleotide (NAD^+^)-dependent deacetylase and involves in stress resistance, apoptosis, senescence, aging, and inflammation [13, 15]. Evidences gathered from a variety of species have indicated that resveratrol provides certain benefits by activating Sirt1, such as improving mitochondrial function [15] and ameliorating both aging [14] and metabolic disorders [39]. In the present study, the protein expression of Sirt1 was increased by dietary resveratrol, in accord with other studies in humans [40], monkeys [39] and rats [15]. In addition, studies have reported that resveratrol relieves oxidative stress induced by H_2_O_2_ [41] and cigarette smoke [42] via Sirt1 activation. In addition, Sirt1 has been shown to be associated with Nrf2 protein stability and expression. Studies have shown that Sirt1 overexpression promotes the nuclear accumulation, DNA binding and transcriptional activity of Nrf2 and increases Nrf2-mediated genes expression [43]. Therefore, activation of the Keap1-Nrf2 pathway may be associated with the increased Sirt1 protein expression.

Oxidative stress, defined as an imbalance between the production of free radicals and reactive metabolites, is closely related to inflammation. Oxidative stress could lead to chronic inflammation, which in turn could mediate many chronic diseases, including cancer, diabetes, and cardiovascular, neurological, and pulmonary diseases [44]. In the present study, we measured pro-inflammatory cytokines in the placenta, but no significant differences were identified in IL-1β, IL-8 or TNF-α levels between Con and Res treatments. However, IL-6 levels in placenta tended to increase with the Res treatment compared with the levels measured for the Con treatment. Accumulating studies have shown that resveratrol displays beneficial activity against inflammatory responses induced by TNF-α [45], lipopolysaccharides [46] and various diseases [17, 36]. However, we failed to observe that pro-inflammatory cytokines levels were influenced by dietary resveratrol. Next, we measured the NFκB-p65 pathway in placenta. We also failed to observe any effect of dietary resveratrol supplementation on NFκB-p65 protein expression. Interestingly, phosphorylated NFκB-p65 protein expression was increased by dietary resveratrol supplementation. NFκB-p65 is responsible for the transcriptional activity of the NFκB complex, which is activated in response to a variety of extracellular signals, such as inflammatory cytokines, infections and multiple stress situations [47]. In unstimulated cells, NFκB is bound to an inhibitory protein, IκB. Binding to IκB masks the nuclear localization signal of NFκB, sequesters the NFκB-IκB complex in the cytoplasm, and prevents NFκB from binding to DNA [34]. Some studies have reported that resveratrol blocks the NFκB pathway or interferes with its transcriptional activity [48, 49]. In addition, resveratrol has been shown to modulate the deacetylation of NFκB via Sirt1 activation and to regulate TNF-α-induced inflammation in human chondrocytes [50]. However, in our study, phosphorylated NFκB-p65 protein expression was increased by dietary resveratrol. If resveratrol can activate the NFκB signaling pathway, we might suppose that the transcription of genes controlled by these transcription factors is also increased. However, mRNA expression of pro-inflammatory genes, including *IL-6*, *IL-1β* and *TNF-α*, did not differ between treatments. An interesting finding is that dietary resveratrol significantly reduced *IL-8* mRNA expression in placenta. Similarly, in human endothelial cells, resveratrol alone or in association with TNF-α induces an increased nuclear appearance of NFκB proteins after overnight incubation [51]. Therefore, we suppose that the increased phosphorylated NFκB-p65 protein expression observed in our study might be beneficial to cells. We conclude that the increase in phosphorylated NFκB-p65 might be insufficient to induce gene transcription.

In the present study, the GSH-Px and CAT activities in plasma of newborn piglets were increased by maternal dietary resveratrol supplementation. The newborn piglets in our study were slaughtered immediately after delivery without colostrum sucking, which indicated that the antioxidant status of newborn piglets may be improved in utero. Resveratrol has been demonstrated to be capable of crossing the placenta and affecting the fetus directly [52]. Therefore, resveratrol not only affects mother but also crosses the placenta and directly affects the fetus during gestation. After birth, pulmonary respiration and a rapid shift from the intrauterine to the hyperoxic extrauterine environment make the newborn vulnerable to the negative effects of oxidative stress, which potentially can impair neonatal vitality [53, 54]. In addition, the vitality of newborn piglets is associated with sucking colostrum, which is important for growth and development. During lactation, colostrum and milk not only provide nutritional and immunological resources but also contain antioxidant enzymes, such as SOD, CAT and GSH-Px, which provide antioxidant protection to infants and newborns. In the present study, the antioxidant enzyme activity in milk was partially increased by dietary resveratrol supplementation. In addition, colostrum contains higher SOD and GSH-Px activity than milk does, which further protects newborns. In the present study, dietary resveratrol showed interaction effects with day of lactation on SOD and GSH-Px activity and MDA level in milk. The CAT activity in milk from sows in the Con treatment reached a peak at d 14 of lactation, whereas that of sows fed Res diets reached the first peak at d 7 of lactation and reached another peak at the end of lactation. In our study, the litter weaning weight and piglet weaning weight in the Res treatment group was higher than those in the Con treatment group, which indicated that reproductive performance was improved by dietary supplementation of resveratrol during gestation and lactation in sows. The improvement of antioxidant enzyme activity in colostrum and milk is beneficial to piglets, which contributes to the antioxidant status of piglets and thus increases the daily weight gain of piglets from birth to weaning [9, 55]. Evidence has shown that supplementation with antioxidants, such as selenium, vitamin E and vitamin C, improves antioxidant status and reproductive performance in sows [11, 55, 56]. Similarly, a growing number of studies have demonstrated that certain functional substances, such as isoflavone [9], ginger extract [57] and chitosan [58], alleviate oxidative stress and improve the reproductive performance of sows. Our results are in accord with these studies and demonstrate that dietary resveratrol exerts beneficial role on the antioxidant defense capacity and reproductive performance of sows.

## Conclusions

In conclusion, our results showed that dietary resveratrol supplementation during gestation and lactation of sows increased the antioxidant status in placenta and milk and thereby increased the antioxidant status of piglets. Dietary resveratrol increased antioxidant gene expression by the Keap1-Nrf2 pathway and Sirt1 in placenta. In addition, dietary resveratrol supplementation increased the litter weaning weight and piglet weaning weights.

## Abbreviations

BW: Body weight
Con: Control treatment
CYP1A1: Cytochrome P450 family 1 subfamily A member 1
GCLM: Glutamate-cysteine ligase modifier
GPx: Glutathione peroxidase
HO1: Heme oxygenase 1
IL-1β: Interleukin 1β
IL-6: Interleukin 6
IL-8: Interleukin 8
IκB: Inhibitor κB
MGST1: Microsomal glutathione S-transferase 1
NFκB: Nuclear factor kappa B
NQO1: NAD(P)H quinone dehydrogenase 1
Nrf2: Nuclear factor E2-related factor 2
Res: Resveratrol treatment
Sirt1: Sirtuin 1
TNF-α: Tumor necrosis factor α
TXNRD1: Thioredoxin reductase 1
UGT1A1: UDP glucuronosyl-transferase family 1 member A1
